# Effect of Prancing and Galloping Drills on Fitness and Ankle Joint Stability in Volleyball Players: A Randomized Trial

**DOI:** 10.1002/jfa2.70090

**Published:** 2025-10-11

**Authors:** Shubham Choudhary, Ankita Sharma, Zoya Zaidi, Waqas Sami, Moattar Raza Rizvi

**Affiliations:** ^1^ Department of Physiotherapy School of Allied Health Sciences Manav Rachna International Institute of Research & Studies (MRIIRS) Faridabad India; ^2^ Department of Physiotherapy Amity Institute of Health Allied Sciences Amity University, Uttar Pradesh Noida India; ^3^ Physiocentric Clinic for Sports Injury Management Manual & Exercise Therapy, Gulmohar Park New Delhi India; ^4^ Department of Pre‐Clinical Affairs College of Nursing QU Health Qatar University Doha Qatar; ^5^ Faculty of Allied Health Sciences Santosh Deemed to be University Ghaziabad Delhi NCR India

**Keywords:** ankle stability, dynamic balance, galloping drills, injury prevention, prancing drills, volleyball

## Abstract

**Introduction:**

Volleyball is a sport that requires rapid changes in movements and high‐intensity actions and skills. Ankle stability is a key factor in preventing injuries and enhance performance in volleyball players. This study evaluated the effects of prancing and galloping drills on ankle stability, agility, and fitness parameters in volleyball players.

**Methods:**

Forty‐two male volleyball players (mean age 23.67 ± 3.15 years) were randomly assigned to the prancing and galloping drills (POGO) training (PGT, *n* = 21) or conventional training (CT, *n* = 21) group. Over 6 weeks, the experimental group performed prancing and galloping drills, whereas the control group continued their routine warm‐up. Outcome measures included ankle stability (Prokin system), vertical jump height (VJH), agility (CODAT), endurance (Cooper test), and dynamic balance (SEBT), assessed after implementing conventional and POGO drills.

**Results:**

Significant improvements were observed in both groups, but the POGO training group demonstrated larger effects. Notably, the POGO training group showed substantial improvements in ankle instability (right ankle: *t* = 4.09 and *p* < 0.001; left ankle: *t* = 5.04 and *p* < 0.001) and dynamic balance (SEBT Right A: *t* = −4.20 and *p* < 0.001; SEBT Left A: *t* = −4.35 and *p* < 0.001).

**Conclusions:**

Prancing and galloping drills effectively enhanced ankle stability and dynamic balance, offering a valuable addition to volleyball training programs for injury prevention and performance enhancement.

## 1 Introduction

Volleyball is a dynamic team sport that demands a combination of explosive power, agility, and precise coordination, particularly during jumping, landing, and rapid directional changes. These high‐intensity biomechanical demands place significant stress ankle joint, making ankle injuries among the most common in volleyball [1]. The landing phase, especially after blocking or spiking, has been identified as a critical moment of vulnerability due to poor joint alignment, limited proprioception, and delayed neuromuscular responses [2]. Ankle stability relies on an integrated system of passive structures (ligaments and joint capsules), active muscle control (e.g., peroneals and tibialis posterior), and sensory feedback mechanisms (proprioceptors) [3, 4]. The neuromuscular coordination refers to the ability of the nervous system to recruit, synchronize, and maintain the joint in its stable position during dynamic tasks [5]. Inadequate neuromuscular coordination and proprioception can reduce the body's ability to detect joint position sense, thus impairing the dynamic joint stability, increasing the risk of both initial and recurrent ankle injuries [6, 7]. Given these concerns, volleyball training protocols frequently incorporate plyometric and proprioceptive exercises to enhance jump performance and reduce injury risks [8]. Plyometric drills involve high‐impact linear movements that focus on vertical force production, whereas proprioceptive exercises often emphasize static balance on unstable surfaces, offering limited carryover to the dynamic multidirectional nature of volleyball [9].

Recent interest has emerged in movement‐based drills, such as prancing and galloping, which may bridge this gap [10, 11]. These low‐impact rhythm‐based exercises combine neuromuscular patterning, proprioceptive feedback, and asymmetrical limb loading, promoting multiplanar control and reflexive joint stabilization. Prancing involves rhythmic elastic spring‐like movements that mimic the eccentric–concentric phases of a jump, potentially improving joint stiffness modulation and timing. Galloping, on the other hand, involves unilateral drive and asymmetric coordination, replicating lateral transition phases during sport‐specific movements [12]. A recent pilot study demonstrated promising improvements in vertical jump height and change of direction performance among volleyball players who underwent a short program of prancing and galloping drills (POGO), supporting their potential for neuromuscular conditioning [10].

Despite these encouraging findings, the application of prancing and galloping as part of structured neuromechanical training programs in volleyball remains underexplored. Existing literature does not show their potential effects on functional parameters, such as dynamic balance, agility, muscular power, or endurance, nor do current studies compare them against conventional warm‐up routines in a randomized design.

Therefore, the present study aimed to investigate the effects of a 6‐week movement‐based training program comprising prancing and galloping drills on ankle stability, agility, vertical jump height, endurance, and dynamic balance in male volleyball players. Male athletes were deliberately selected because sex‐related differences in neuromuscular control, ligamentous laxity, and ankle injury incidence could otherwise confound outcomes in a relatively small sample, consistent with SAGER guideline recommendations [13]. By comparing this protocol, the study seeks to determine whether these underutilized coordination‐driven drills can serve as an effective and joint‐protective alternative in competitive training environments [10].

## 2 Materials and Methods

### 2.1 Study Design, Setting, and Duration

This study adopted a randomized controlled trial (RCT) design to examine the effects of prancing and galloping drills (POGO training) on ankle stability and physical performance among volleyball players. Participants were randomly assigned to either the POGO training group (PGT, *n* = 26) or the conventional training group (CT, *n* = 26) using a computer‐generated sequence. Allocation concealment was ensured through the use of sealed opaque envelopes prepared by an independent researcher. Both the participants and outcome assessor were blinded to the group allocation; the outcome assessor was not a part of the training delivery. The trial was conducted at the Manav Rachna Sports Science Center and the Late Prannath Volleyball Academy. The intervention period lasted 6 weeks, with outcome assessments conducted at baseline and following the intervention.

### 2.2 Sample Size

**FIGURE 1:**
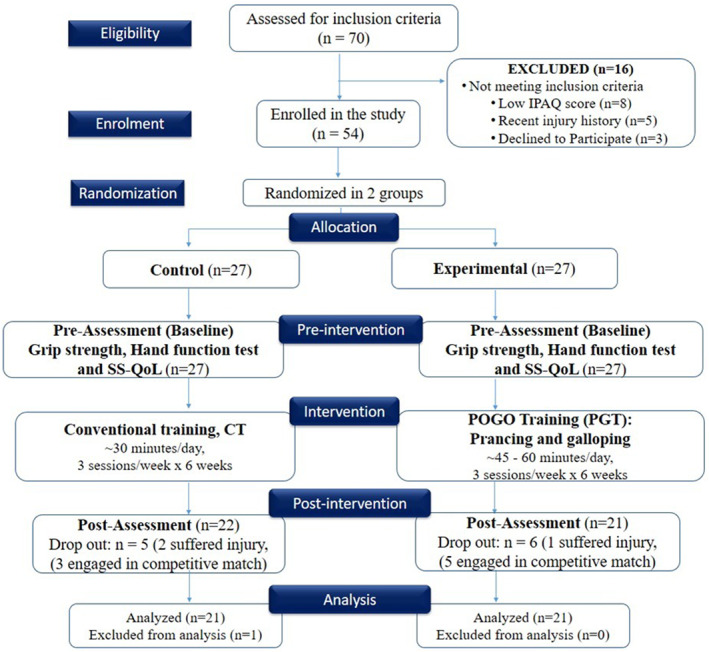
The PRESENT (Proper Reporting of Evidence in Sport and Exercise Nutrition Trials) 2020 checklist guidelines depict the flow of participants through a trial, including enrollment, allocation, follow‐up, and analysis.

Sample size estimation was conducted using G*Power version 3.1.9.4, targeting a repeated‐measures ANOVA (within–between interaction) design with two groups and two time points (preintevention and postintervention). Using a medium effect size (Cohen's *f* = 0.25), alpha level (*α* = 0.05), power (1 – *β* = 0.95), correlation among repeated measures (*r* = 0.5), and nonsphericity correction (*ε* = 1), the minimum total sample size required was 54 participants (*n* = 27 per group). A total of 70 volleyball players were screened, of whom 54 met the eligibility criteria and were enrolled in the study (Figure 1).

### 2.3 Sampling Method

A convenience sampling approach was used to recruit participants. The sample comprised male volleyball athletes competing at the district level or higher. Male‐only recruitment was chosen because documented sex‐based differences in ankle joint stability and neuromuscular coordination could introduce heterogeneity in a limited sample size. Additionally, organized men's teams were more accessible in the recruitment setting, ensuring consistent delivery of the intervention. The study design and reporting followed the SAGER (Sex and Gender Equity in Research) guidelines, with an evidence‐informed rationale provided for focusing on male participants [13].

### 2.4 Inclusion and Exclusion Criteria

Participants were eligible if they were male volleyball players aged 18–26 years, competing at district‐level tournaments or above. All participants were required to engage in regular volleyball training for a minimum of 200 min per week for at least 6 months. Moderate‐to‐high activity levels (MET scores between 600 and 1500) were included were verified using the International Physical Activity Questionnaire (IPAQ) [14]. Athletes with a history of lower limb injuries in the preceding 6 months were excluded.

### 2.5 Ethical Approval

Ethical clearance was obtained from the Institutional Ethics Committee at the Faculty of Allied Health Sciences, Manav Rachna International Institute of Research and Studies (Reference No.: MRIIRS/FAHS/PT/2022‐23/S‐19; dated January 9, 2023), in accordance with the ethical principles of the Declaration of Helsinki. The study was prospectively registered with the Clinical Trials Registry–India (CTRI/2023/05/052815).

### 2.6 Procedure

Prior to enrollment, participants were thoroughly briefed on the procedures, potential risks, and benefits of the study and provided informed written consent. The intervention was delivered under ethical and athlete‐centered guidelines.

The conventional training [15] group continued their standard warm‐up routines, which included dynamic and static stretching exercises followed by volleyball‐specific skill drills. Dynamic movements, such as arm circles, leg swings, high knees, butt kicks, lateral shuffles, cariocas, and jumping jacks, were used to activate major muscle groups. Static stretching involves exercises for the quadriceps, hamstrings, calves, gluteals, and shoulders. Skill‐based drills were routinely practiced by the team under the supervision of their coach 3 times a week for 6 weeks.

**FIGURE 2:**
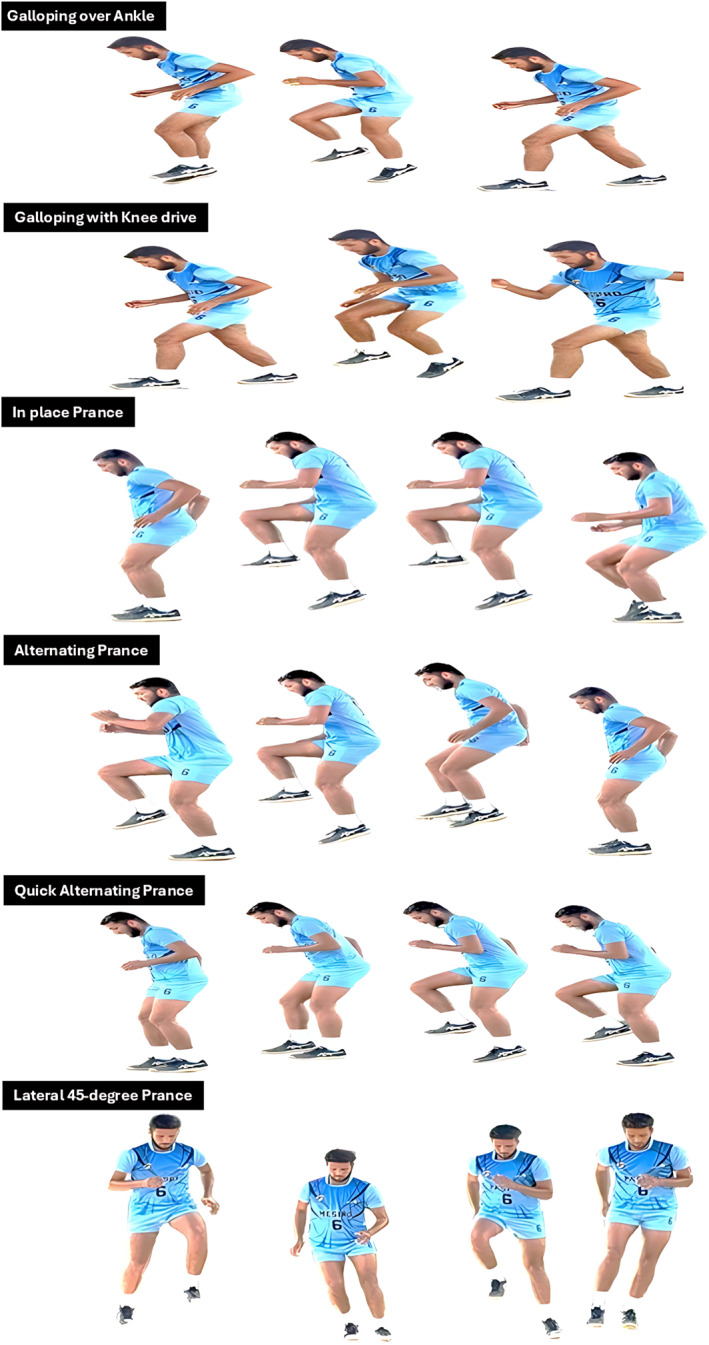
Prancing and galloping drills, including galloping over ankle and with knee drive, in place prance, alternating prance, quick alternating prance, and lateral 45‐degree prance.

In contrast, the POGO training (PGT) group incorporated a structured sequence of prancing and galloping drills into their warm‐up routine, conducted three times per week over six weeks, resulting in a total of 18 supervised sessions (Table 1, Figure 2), performed on an indoor synthetic volleyball court (Taraflex surface, 18 × 9 m) under controlled temperature (22°C–24°C), with athletes wearing standard non‐marking volleyball shoes. No additional equipment was used apart from floor markers to indicate movement zones. These drills were selected for their biomechanical relevance to volleyball, particularly their ability to stimulate neuromuscular coordination, proprioceptive responsiveness, and dynamic joint stabilization during asymmetric and multiplanar movements. The intervention was delivered in two progressive phases. Phase 1, spanning weeks 1–3, focused on developing foundational neuromechanical skills such as rhythm, balance, postural control, and movement symmetry. Each session lasted approximately 20 min, with each drill performed for 30–45 s followed by 15–20 s of rest, repeated for 3–4 sets. Exercise intensity was targeted at a perceived exertion of 5–6 (“hard”) on the Borg CR‐10 scale.

**TABLE 1:** Prancing and galloping training protocol for volleyball players (Figure 2).

Drill type	Drill name	Description	Reps/distance/time
Weeks 1–3 (level 1): Foundation and coordination			
Prancing drills	In‐place prance	Perform rhythmic prancing in a stationary position, emphasizing interlimb coordination and postural control	3 sets, 15 s
	Alternating prance	Perform prancing with alternating leg drives to simulate forward propulsion and coordination	3 sets, 40 m
	Quick alternating prance	Execute rapid alternating steps with vigorous arm swing to enhance reactivity and neuromotor speed	3 sets, 40 m
Galloping drills	Galloping drill over ankle	Perform low‐amplitude galloping to reinforce ankle stability and frontal plane control	3 sets, 40 m
	Galloping drill with knee drive	Integrate a high knee drive to promote hip flexor engagement and vertical force generation	3 sets, 40 m
Weeks 4–6 (level 2): Advanced strength and agility			
Prancing drills	In‐place prance (progressed)	Increase rhythm complexity and muscular control through enhanced speed and range	4 sets, 15 s
	Alternating prance (progressed)	Increase leg drive intensity and refine arm‐leg synchronization to boost propulsion	4 sets, 40 m
	Quick alternating prance (progressed)	Maximize speed and force production through explosive short‐contact ground interactions	4 sets, 40 m
	Lateral 45‐degree prance	Perform alternating prancing at a 45° lateral angle to develop multidirectional agility	4 sets, 40 m
Galloping drills	Galloping drill over ankle (progressed)	Increase movement velocity while maintaining ankle‐focused control and limb dissociation	4 sets, 40 m
	Galloping drill with knee drive (progressed)	Enhance explosive force by integrating higher amplitude knee drives under speed	4 sets, 40 m
	Hurdle gallops	Gallop over low hurdles placed at 5‐m intervals, alternating lead legs to reinforce dynamic stability and spatial accuracy	4 sets, 40 m

Phase 2, implemented during weeks 4–6, introduced more advanced drills targeting dynamic stability, directional reactivity, eccentric–concentric force control, and explosive transitions, delivered in small groups of 4–6 players with live demonstration before each exercise and real‐time verbal feedback during performance. Players executed drills simultaneously but received individualized corrections when deviations from correct form were observed. All sessions were supervised by the same licensed physiotherapist (Master's in Sports Physiotherapy with 5 years of experience in volleyball performance conditioning) to ensure consistent delivery and adherence to the protocol. Participants in both groups continued their regular volleyball training under the supervision of their team coach. Motivation strategies included verbal encouragement, peer comparison challenges, and positive reinforcement based on execution quality scores. Progressions were adjusted individually according to baseline ankle stability scores: athletes with instability performed reduced amplitude gallops, whereas higher performers progressed to faster tempos and greater knee drive. The intervention description follows the Consensus on Exercise Reporting Template (CERT) guidelines to ensure comprehensive and transparent reporting [16].

### 2.7 Outcome Measures and Assessment Protocol

All assessments were conducted at baseline and after the 6‐week intervention period. Two trained physiotherapists, blinded to group allocation and uninvolved in the intervention delivery, performed all measurements to reduce assessor bias. The testing was conducted in a controlled indoor environment with uniform surface conditions, lighting, and temperature to ensure procedural consistency.

### 2.8 Ankle Proprioception and Stability

Ankle proprioception and static postural stability were assessed using the Prokin System (TechnoBody, Italy), a validated computerized platform for measuring balance and sensorimotor performance. This system has been shown to have good reliability with single‐leg stability testing [17]. Participants performed a single‐leg stance for both legs alternatively on an unstable footboard while attempting to keep a cursor centered on a screen. The dominant leg was assessed first, followed by the nondominant leg, and the same sequence was applied across both groups. The system recorded center‐of‐pressure deviations, which reflect proprioceptive accuracy and neuromuscular control under unstable conditions. The Prokin has been used extensively in previous studies examining lower‐limb sensorimotor training outcomes [18].

### 2.9 Dynamic Balance

Dynamic balance was evaluated using the Star Excursion Balance Test (SEBT) for both legs. Participants stood on one leg and reached to all eight directions to the stance limb (anterior, anteromedial, medial, posteromedial, posterior, posterolateral, lateral, and anterolateral) using the opposite limb, while maintaining postural stability. Both the legs were assessed for balance among the groups, dominant followed by the nondominant. SEBT is known for its sensitivity in detecting side‐to‐side asymmetries and neuromotor deficits related to ankle instability [19].

### 2.10 Explosive Lower‐Limb Power

Explosive power was assessed using the Sargent vertical jump test, which required participants to perform maximal vertical jumps from a standing position. The jump height was measured using a calibrated wall‐mounted scale. This test provides a reliable and valid estimate of lower‐extremity muscular strength and power output [20]. Vertical jumping ability is a key performance metric in volleyball due to its relevance to blocking, spiking, and overhead reach mechanics [21].

### 2.11 Agility and Change‐of‐Direction Ability

Agility performance was measured using the change‐of‐direction ability test (CODAT). Participants were instructed to sprint through a standardized course involving lateral and diagonal transitions, with time to completion recorded using a stopwatch. The test‐retest reliability for CODAT is found to be high (ICC = 0.84; variation coefficient = 3%), which makes it a test of choice for measuring neuromotor reactivity and directional speed [22].

### 2.12 Aerobic Capacity

Aerobic endurance was assessed using the 12‐min Cooper run test, which required participants to run continuously on a marked indoor track [23]. The total distance covered was recorded in meters and used to estimate aerobic fitness levels. This test remains a reliable indicator of cardiovascular endurance and has been previously validated in athletic populations [24].

### 2.13 Statistical Analysis

Statistical analyses were conducted using IBM SPSS Statistics for Windows, version 22.0 (IBM Corp., Armonk, NY, USA). The Shapiro–Wilk test was used to verify the normality of continuous data distributions. For between‐group comparisons at baseline and postintervention, independent samples *t*‐tests were employed. To assess within‐group changes over time, paired samples *t*‐tests were conducted. To evaluate both time and group effects concurrently, a two‐way mixed‐model analysis of variance (2 × 2 ANOVA) was performed. This model included one within‐subjects factor (time: preintevention and postintervention) and one between‐subjects factor (group: PGT vs. CT), allowing examination of main effects for time, main effects for group, and time × group interaction effects. In addition to significance testing, partial eta squared (*η* ^2^ *p*) was computed as a measure of effect size, indicating the proportion of variance in the dependent variable attributable to a given factor while accounting for other sources of variance. Interpretation thresholds followed Cohen's convention, where values of 0.01, 0.06, and 0.14 were considered small, medium, and large effects. Descriptive statistics are presented as means ± standard deviations (SD). A *p*‐value of ≤ 0.05 was considered statistically significant across all tests.

## 3 Results

### 3.1 Participant Flow and Baseline Characteristics

Out of the 54 participants initially enrolled in the study, 27 were allocated to the POGO training (PGT) group and 27 to the conventional training [15] group. During the six‐week intervention, 11 participants discontinued participation. The CT group experienced five dropouts, two due to injury and three due to scheduling conflicts with competitive matches, wheeas the PGT group reported six dropouts, with one injury‐related and five match‐related exclusions. Consequently, 21 participants from each group completed the study and were included in the final analysis (Figure 1). In the PGT group, mean attendance across the 18 scheduled sessions was 94%, with 95% of sets rated as “acceptable” or “excellent” by the supervising physiotherapist. No adverse events (falls, sprains, or prolonged soreness) were reported during the intervention.

### 3.2 Baseline Group Comparisons (Independent *t*‐tests)

Baseline comparisons confirmed that the two groups were statistically comparable across all demographic and performance variables. The mean age and SD in the CT group were 23.67 ± 3.15 years and 24.67 ± 3.07 years in the PGT group (*t* = −1.04 and *p* = 0.30). Body mass index (BMI) values were also similar between groups (CT: 22.26 ± 2.20 kg/m^2^; PGT: 22.39 ± 2.49 kg/m^2^; and *t* = −0.18 and *p* = 0.86).

Explosive power, measured by vertical jump height (VJH), was comparable between CT (50.43 ± 3.75 cm) and PGT (50.38 ± 6.48 cm; *t* = 0.03 and *p* = 0.98). Agility (CODAT) was also similar between groups (CT: 5.86 ± 0.19 s; PGT: 5.88 ± 0.36 s; and *t* = −0.26 and *p* = 0.80). Ankle instability scores (Prokin system) revealed no significant differences between CT and PGT for the right (0.98 ± 0.16 vs. 0.99 ± 0.17; *t* = −0.10 and *p* = 0.92) or left ankle (1.19 ± 0.22 vs. 1.31 ± 0.48; *t* = −1.08 and *p* = 0.29). Endurance (Cooper test) was slightly higher in the PGT group (2.61 ± 0.14 km) than the CT group (2.53 ± 0.23 km), but the difference was not statistically significant (*t* = −1.45, *p* = 0.15). Dynamic balance, measured by the SEBT, showed no significant baseline differences across all eight directions for both right and left limbs. Full details of these comparisons are provided in Table 2.

**TABLE 2:** Within‐group (paired *t*‐test) and between‐group (repeated measures ANOVA) comparisons of power, agility, ankle instability, and endurance.

Outcome measures	Group	Paired differences	95% CI lower, upper	*t*	*p*	Time effect	Group effect	Time × group interaction
						(*p*, *η* ^2^ *p*)	(*p*, *η* ^2^ *p*)	(*p*, *η* ^2^ *p*)
Power (VJH)	CT	−1.86	−2.98, −0.74	−3.47	< 0.001	< 0.001 (0.71)	0.24 (0.03)	< 0.001 (0.38)
	PGT	−5.62	−6.72, −4.52	−10.65	< 0.001			
Agility (CODAT)	CT	−0.19	−0.28, −0.10	−4.49	< 0.001	< 0.001 (0.71)	< 0.001 (0.16)	< 0.001 (0.41)
	PGT	−0.62	−0.77, −0.48	−8.91	< 0.001			
Ankle instability right	CT	−0.04	−0.13, 0.05	−0.96	0.35	0.06 (0.08)	0.03 (0.12)	< 0.001 (0.22)
	PGT	0.14	0.07, 0.22	4.09	< 0.001			
Ankle instability left	CT	−0.05	−0.13, 0.03	−1.28	0.22	< 0.001 (0.26)	0.41 (0.02)	< 0.001 (0.38)
	PGT	0.34	0.20, 0.49	5.04	< 0.001			
Endurance (cooper test)	CT	−0.29	−0.37, −0.20	−6.69	< 0.001	0.25 (0.03)	0.05 (0.09)	0.25 (0.03)
	PGT	−0.36	−0.45, −0.27	−8.23	< 0.001			

Abbreviations: CI, confidence interval; CT, conventional training; PGT, POGO training; *p*, significance value; *t*, statistical value for paired *t* test; *η* ^2^ *p*, partial eta square (effect size).

### 3.3 Within‐Group Improvements (Paired *t*‐Test Analysis)

Significant pre–post improvements were observed within both groups for several outcome measures (Table 2). In the CT group, vertical jump height improved significantly (*t* = −3.47 and *p* < 0.001) as did agility (mean change: −0.19 s; *t* = −4.49 and *p* < 0.001) and endurance (mean change: +0.29 km; *t* = −6.69 and *p* < 0.001). No significant changes were found in ankle instability scores.

In contrast, the PGT group exhibited significantly greater within‐group improvements. Vertical jump height increased substantially (*t* = −10.65 and *p* < 0.001), and agility improved by a mean of −0.62 s (*t* = −8.91 and *p* < 0.001). Ankle instability scores improved significantly for both the right (*t* = 4.09 and *p* < 0.001) and left (*t* = 5.04 and *p* < 0.001) ankles. Endurance also improved (mean change: +0.36 km; *t* = −8.23 and *p* < 0.001).

SEBT reach distances improved significantly in the PGT group across nearly all directions for both limbs (Table 3). For the right leg, significant changes were found in the anterior (*p* < 0.001), anteromedial (*p* = 0.001), medial (*p* = 0.001), posteromedial (*p* = 0.002), posterior (*p* < 0.001), lateral (*p* = 0.001), and anterolateral (*p* < 0.001) directions. For the left leg, improvements were also observed in the anterior (*p* < 0.001), anteromedial (*p* = 0.002), medial (*p* = 0.001), posterior (*p* = 0.001), lateral (*p* < 0.001), and anterolateral (*p* < 0.001) directions.

**TABLE 3:** Within‐group (paired *t*‐test) and between‐group (repeated measures ANOVA) comparisons of dynamic balance of right leg and left leg.

Outcome measures	Group	Paired differences	95% CI lower, upper	*t*	*p*	Time effect	Group effect	Time × group interaction
						(*p*, *η* ^2^ *p*)	(*p*, *η* ^2^ *p*)	(*p*, *η* ^2^ *p*)
SEBT right A	CT	−2.60	−3.79, −1.41	−4.57	< 0.001	< 0.001 (0.4)	0.44 (0.51)	0.004 (0.2)
	PGT	−10.60	−15.89, −5.31	−4.20	< 0.001			
SEBT right AM	CT	−1.38	−3.99, 1.24	−1.10	0.284	< 0.001 (0.28)	0.26 (0.03)	0.01 (0.16)
	PGT	−7.74	−11.94, −3.53	−3.85	0.001			
SEBT right M	CT	−0.67	−3.80, 2.47	−0.44	0.662	< 0.001 (0.26)	0.46 (0.01)	0.003 (0.21)
	PGT	−9.32	−14.05, −4.59	−4.12	0.001			
SEBT right PM	CT	−0.64	−4.55, 3.27	−0.34	0.736	< 0.001 (0.2)	0.48 (0.01)	0.01 (0.16)
	PGT	−8.96	−14.06, −3.86	−3.68	0.002			
SEBT right P	CT	1.24	−2.85, 5.32	0.63	0.535	< 0.001 (0.3)	0.63 (0.01)	0.02 (0.21)
	PGT	−12.03	−17.47, −6.58	−4.63	< 0.001			
SEBT right PL	CT	−2.42	−4.78, −0.05	−2.14	0.046	< 0.001 (0.3)	0.06 (0.002)	0.006 (0.18)
	PGT	−11.26	−17.20, −5.31	−3.96	0.001			
SEBT Right L	CT	−1.85	−4.62, 0.93	−1.39	0.179	< 0.001 (0.33)	0.3 (0.58)	0.003 (0.21)
	PGT	−11.69	−17.55, −5.83	−4.18	0.001			
SEBT right AL	CT	−2.02	−3.94, −0.10	−2.20	0.040	< 0.001 (0.38)	0.9 (0.7)	0.002 (0.22)
	PGT	−10.56	−15.66, −5.45	−4.33	< 0.001			
SEBT left A	CT	−0.17	−2.41, 2.08	−0.15	0.879	< 0.001 (0.2)	0.43 (0.51)	0.001 (0.27)
	PGT	−8.77	−12.99, −4.54	−4.35	0.000			
SEBT left AM	CT	0.27	−3.01, 3.54	0.17	0.867	< 0.001 (0.19)	0.4 (0.5)	0.003 (0.21)
	PGT	−9.90	−15.67, −4.13	−3.59	0.002			
SEBT right M	CT	−1.46	−5.64, 2.72	−0.73	0.474	< 0.001 (0.2)	1.3 (0.2)	0.01 (0.13)
	PGT	−11.25	−17.32, −5.17	−3.87	0.001			
SEBT left PM	CT	−2.26	−4.73, 0.22	−1.91	0.072	< 0.001 (0.03)	0.03 (0.86)	0.01 (0.15)
	PGT	−9.32	−14.43, −4.20	−3.81	0.001			
SEBT left P	CT	−0.32	−3.77, 3.14	−0.19	0.851	< 0.001 (0.2)	0.001 (0.97)	0.002 (0.22)
	PGT	−10.52	−15.94, −5.09	−4.06	0.001			
SEBT left PL	CT	−1.32	−4.81, 2.17	−0.79	0.438	< 0.001 (0.16)	0.2 (0.86)	0.001 (0.1)
	PGT	−10.02	−17.85, −2.18	−2.68	0.015			
SEBT Left L	CT	−0.94	−3.51, 1.64	−0.76	0.456	< 0.001 (0.3)	0.7 (0.7)	0.002 (0.3)
	PGT	−13.11	−18.54, −7.68	−5.06	< 0.001			
SEBT left AL	CT	−2.21	−5.00, 0.58	−1.66	0.114	< 0.001 (0.3)	0.25 (0.87)	0.003 (0.2)
	PGT	−11.32	−16.67, −5.97	−4.43	< 0.001			

Abbreviations: A, anterior; AL, anterolateral; AM, anteromedial; CI, confidence interval; CT, conventional training; L, lateral; M, medial; P, posterior; PGT, POGO training; PL, posterolateral; PM, posteromedial; *p*, significance value; *t*, statistical value for paired *t* test; *η* ^2^ *p*, partial eta square (effect size).

### 3.4 Between‐Group Effects and Interaction Effects (Mixed‐Model ANOVA)

Repeated measures ANOVA revealed significant main effects of time for all outcome measures (*p* < 0.001), confirming that both groups improved over the intervention period. However, group effects and time × group interaction effects further highlighted the superior performance gains in the PGT group (Table 2). Significant between‐group effects were observed in agility (*p* < 0.001 and *η* ^2^ *p* = 0.41), ankle instability for the right (*p* < 0.001 and *η* ^2^ *p* = 0.22) and left (*p* < 0.001 and *η* ^2^ *p* = 0.38) limbs, and endurance (*p* = 0.05 and *η* ^2^ *p* = 0.09). Significant time × group interactions were observed for vertical jump height (*p* < 0.001 and *η* ^2^ *p* = 0.38), agility (*p* < 0.001 and *η* ^2^ *p* = 0.41), and ankle instability (right: *p* < 0.001 and *η* ^2^ *p* = 0.22; left: *p* < 0.001 and *η* ^2^ *p* = 0.38), indicating that the PGT group experienced greater gains across all core functional outcomes compared to CT.

### 3.5 SEBT Between‐Group and Interaction Effects

In terms of dynamic balance, repeated measures ANOVA (Table 3) showed significant time × group interactions for the right anterior (*p* = 0.004 and *η* ^2^ *p* = 0.20) and left anterior (*p* = 0.001 and *η* ^2^ *p* = 0.27) directions, indicating that improvements were significantly greater in the PGT group. Significant group effects were also found for the right anteromedial (*p* = 0.01 and *η* ^2^ *p* = 0.16), right posterior (*p* = 0.02 and *η* ^2^ *p* = 0.21), and left anterior (*p* = 0.001 and *η* ^2^ *p* = 0.27) directions. Additional interaction effects were noted for right medial (*p* = 0.003 and *η* ^2^ *p* = 0.21), left lateral (*p* = 0.003 and *η* ^2^ *p* = 0.21), and left anterolateral (*p* = 0.003 and *η* ^2^ *p* = 0.20).

## 4 Discussion

The primary aim of this study was to evaluate the effectiveness of prancing and galloping drills in enhancing ankle stability, agility, and physical performance in volleyball players. The results showed that the prancing and galloping training (PGT) protocol produced greater improvements than the conventional training [15] group across multiple functional domains, including dynamic balance, explosive power, agility, and ankle proprioception. The rhythmic, asymmetrical, and multiplanar nature of prancing and galloping promotes neuromuscular coordination and joint stability, which are vital for performance and injury prevention [10, 25, 26].

Significant improvements in ankle stability were noted only in the PGT group. These results align with previous research demonstrating superior gains in joint stability and postural control through dynamic plyometric interventions compared to static balance training [20, 27, 28]. The rapid direction changes and asymmetric loading in the PGT drills likely enhanced proprioception [29]. Such proprioceptive improvements have been associated with reduced ankle injury recurrence and better neuromotor control [30]. Additionally, repeated spring‐like movements may have strengthened the peroneal muscles, key stabilizers during cutting and landing in volleyball [31].

Both groups improved in vertical jump height, but the PGT group demonstrated greater gains. This is consistent with studies showing up to 10% improvements in jump performance following plyometric training [20, 32]. Vertical jumping is crucial in volleyball, especially for positions, such as outside and opposite hitters, who require high spike reach, and middle blockers who rely on both spike and block capabilities [33]. The repeated eccentric‐concentric loading in prancing and galloping likely improved lower‐limb explosive strength. Galloping drills, in particular, target unilateral drive and ground‐reaction timing, contributing to spike and block performance [34]. These findings affirm the value of the POGO protocol as a sport‐specific strength and power development tool.

Agility improved in both groups, with significantly greater gains in the PGT group. Training focused on rapid directional changes has been shown to enhance lateral speed and defensive movements in volleyball [35]. Drills, such as shuttle runs and ladder‐based footwork mechanically similar to prancing and galloping, have been effective in boosting agility [26]. Similar improvements were observed following plyometric jump protocols in volleyball athletes [36]. Galloping drills require reactive transitions and unstable loading, enhancing neuromotor readiness. Prancing drills also contributed to agility by developing lower‐limb coordination and control for linear and nonlinear movements [37].

Aerobic endurance, measured by the 12‐min Cooper test, improved significantly in both groups, with superior gains in the PGT group. These findings are supported by prior work showing that high‐intensity repetitive training enhances cardiovascular endurance [38]. The dynamic continuous nature of drills likely contributed to both aerobic and anaerobic adaptations, which are important for maintaining performance during prolonged rallies [39].

Dynamic balance, as assessed by SEBT, improved significantly in both groups, with the PGT group exhibiting greater enhancements. Prior research has shown that plyometric and neuromuscular drills improve postural control, proprioception, and landing force modulation [40]. Dynamic stabilization and jump training protocols have been associated with better balance and lower‐limb force absorption [27]. This was supported by findings that plyometric training outperforms resistance modalities, such as kettlebell exercises, for agility and balance gains [41]. Given the high frequency of multiplanar movements in volleyball, such as spiking, blocking, and side‐stepping, balance training grounded in sport‐specific drills is essential. The present results highlight the potential of POGO training to address this performance demand, with high adherence rates and absence of adverse events indicating that the protocol is both feasible and safe for integration into regular training schedules.

### 4.1 Limitations

This study presents several limitations that must be considered when interpreting the findings. The relatively small and demographically homogeneous sample limits the generalizability of the results. This study included only male volleyball players to minimize sex‐related variability, which could confound results in a relatively small sample. Although this approach enhanced internal validity, it also limits generalizability. However, this choice limits the generalizability of findings to female athletes, who may differ in neuromuscular control, ligamentous laxity, and injury risk. In line with SAGER guideline points 4b and 5b, we explicitly acknowledge this limitation and recommend that future research include female participants or perform sex‐stratified analyses. The absence of long‐term follow‐up precludes assessment of the sustainability and retention of the observed improvements. The potential confounding variables—such as participants' nutritional intake, sleep quality, and involvement in parallel training—were not controlled. Finally, although the prancing and galloping drills offer a novel movement‐based alternative, their superiority over well‐established training approaches, such as plyometric or proprioceptive programs, should be interpreted with caution.

### 4.2 Conclusion

This study demonstrated that prancing and galloping drills significantly improved ankle stability, agility, dynamic balance, and physical fitness in competitive volleyball players. The PGT consistently outperformed the CT group across key performance domains, suggesting that rhythm‐based movement‐driven drills offer a promising alternative to traditional warm‐up protocols. These findings highlight the potential value of integrating multiplanar and proprioceptively rich activities into sport‐specific training regimens, particularly in sports, such as volleyball, that demand frequent jumping, directional changes, and reactive stability.

### 4.3 Future Scope

Future studies should address these limitations by including larger and more diverse samples, incorporating both male and female athletes across varying skill levels. Longitudinal research designs are needed to evaluate the durability of training effects and their influence on injury recurrence. Additionally, combining prancing and galloping drills with other modalities—such as resistance training, flexibility routines, or neuromuscular rehabilitation protocols—could provide a more comprehensive understanding of their role in enhancing athletic function. Finally, the inclusion of biomechanical analyses, such as kinematic or electromyographic assessments, could offer deeper insights into the neuromuscular mechanisms driving these improvements.

## Author Contributions

**Shubham Choudhary:** conceptualization, investigation, methodology. **Ankita Sharma:** conceptualization, resources, writing – original draft. **Zoya Zaidi:** investigation, methodology, supervision. **Waqas Sami:** conceptualization, data curation, formal analysis, supervision, validation, writing – review and editing. **Moattar Raza Rizvi:** conceptualization, data curation, formal analysis, validation, writing – original draft, writing – review and editing.

## Ethics Statement

Ethical approval for the study was obtained from the Ethical Committee at the Faculty of Allied Health Sciences (Reference No.: MRIIRS/FAHS/PT/2022‐23/S‐19, dated January 9, 2023), in accordance with the principles outlined in the Declaration of Helsinki. The trial was registered with the Clinical Trials Registry—India (CTRI) under the registration number CTRI/2023/05/052815.

## Consent

The author confirms that informed consent for the publication of research findings, including photographs, videos, and any personal or identifiable data, was obtained from all participants prior to publication.

## Conflicts of Interest

The authors declare no conflicts of interest.

## Data Availability

The data presented in this study are available from the corresponding author upon request. The data are not publicly available due to privacy restrictions.
